# Role of the tonsil–oropharynx ratio on lateral cephalograms in assessing tonsillar hypertrophy in children seeking orthodontic treatment

**DOI:** 10.1186/s12903-023-03573-z

**Published:** 2023-11-07

**Authors:** Chenxing Lv, Liu Yang, Peter Ngan, Wenjie Xiao, Tingting Zhao, Bojun Tang, Xiong Chen, Hong He

**Affiliations:** 1 State Key Laboratory of Oral and Maxillofacial Reconstruction and Regeneration, Key Laboratory of Oral Biomedicine Ministry of Education, Hubei Key Laboratory of Stomatology, School and Hospital of Stomatology, Wuhan University, Wuhan, China; 2 Department of Orthodontics, School and Hospital of Stomatology, Wuhan University, Wuhan, China; 3 Department of Stomatology, Hangzhou Traditional Chinese Medicine Hospital, Hangzhou, China; 4 Department of Orthodontics, School of Dentistry, West Virginia University, Morgantown, USA; 5 Department of Otorhinolaryngology-Head and Neck Surgery, Guangdong Clifford Hospital, Guangzhou, China; 6 Department of Otorhinolaryngology-Head and Neck Surgery, Zhongnan Hospital of Wuhan University, Wuhan, China

**Keywords:** Tonsillar hypertrophy, Lateral cephalogram, Palatine tonsil size, Children

## Abstract

**Objectives:**

To analyze the diagnostic value of the tonsil–oropharynx (T/O) ratio on lateral cephalograms for evaluating tonsillar hypertrophy (TH).

**Methods:**

A cross-sectional study was performed on 185 consecutive children (101 males, 84 females; mean age 7.3 ± 1.4 years) seeking orthodontic treatment. The T/O ratios on lateral cephalograms were calculated following Baroni et al.’s method. Tonsil sizes were clinically determined according to the Brodsky grading scale. Spearman correlation coefficients between the T/O ratio and clinical tonsil size were calculated with the total sample and subgroups and then compared between subgroups. Diagnostic value was analyzed using the receiver operating characteristic (ROC) curve, sensitivity, specificity, positive and negative predictive values, and accuracy.

**Results:**

There was a strong correlation between the T/O ratio and clinical tonsil size in children (ρ = 0.73; P < 0.001). A significantly higher correlation coefficient was found in the Class III children. The ROC curve revealed an area under the curve of 0.90 (95% CI, 0.86–0.94; P < 0.001). The optimal cutoff value of the T/O ratio for predicting TH was 0.58, with a sensitivity of 98.7% and specificity of 64.2%. Employing the cutoff value of 0.5, the sensitivity was 100% and the specificity was 45.9%.

**Conclusions:**

Measurement of the T/O ratio on lateral cephalograms may be helpful to initial screening in children for TH. Practitioners may combine the clinical examination of tonsil size with the cephalometric findings for a more comprehensive evaluation.

**Supplementary Information:**

The online version contains supplementary material available at 10.1186/s12903-023-03573-z.

## Introduction

The palatine tonsils are lymphatic tissues located on the lateral walls of the oropharynx. The tonsil tissues are very small at birth, usually, enlarge in size throughout childhood, and tend to regress in adolescence [1, 2]. Tonsillar hypertrophy (TH) is common in the pediatric population and may contribute to airway obstruction, the development of obstructive sleep apnea (OSA) as well as abnormal dentofacial growth [3–6].

In recent decades, the association between TH and Class III malocclusion of children has attracted increasing attention [5–9]. The literature suggested that children with isolated TH had a more forward and upward position of the mandible [5, 6] and a higher rate of Class III relationships [7]. Iwasaki et al. [8] depicted that the tonsil size of Class III children was significantly correlated with anterior tongue posture and mandibular protrusion. Therefore, the correct diagnosis of TH should be made before treating respiratory problems and related dentofacial deformities in children.

The current standard grading system for evaluating tonsil size and diagnosing TH is based on clinical oropharyngeal examination [10]. The tonsils are assigned a grade depending on the percentage of oropharyngeal airway occupied by tonsils in the medial-lateral dimension [11]. However, this widely used examination is not perfect as it fails to reveal oropharyngeal obstruction in the anterior-posterior dimension [12–14].

As a standard orthodontic method to evaluate craniofacial morphology, the lateral cephalogram can be a ready reference for orthodontists to evaluate airway obstruction and hypertrophic adenoids and tonsils [6, 15]. Many studies have reported that cephalometric measurement of adenoid had reasonable correlations to adenoid size [15, 16] and lateral cephalogram exhibited good accuracy for the diagnosis of adenoid hypertrophy [17]. Unlike adenoids, there is a lack of enough existing cephalometric guidelines for diagnosing TH, although lateral cephalograms have been used to recognize obstructive tonsils in dental practice [5, 6, 18].

Behlfelt et al. [19] and Pirila-Parkkinen et al. [20] demonstrated that the cephalometric-measured area of tonsil or tonsil and soft palate was positively correlated with clinical tonsil size, but the validity of these parameters in quantifying tonsil size was not analyzed. In contrast to cross-sectional area measurements, Baroni et al. [5] proposed an easy-to-use cephalometric method to identify hypertrophic tonsils using linear measurements. However, the diagnosis of TH was subjectively made based on whether the ratio of oropharyngeal obstruction by tonsils was greater than 0.5. For a simplified description, we define this ratio as the tonsil–oropharynx (T/O) ratio.

As it can be easily applied in both manual tracing and software, the method of calculating the T/O ratio seems more practical and less time-consuming than area measurements to predict clinical tonsil size. However, the accuracy of the cutoff value at 0.5 is not known and no study has yet sought to evaluate its correlation with clinical tonsil size. Therefore, the aims of this study were (1) to investigate the correlation between the cephalometric T/O ratio and clinical (subjective) tonsil size, and (2) to analyze the diagnostic value of the T/O ratio on lateral cephalograms for evaluating TH (using clinical examinations as the reference standard).

## Materials and methods

### Sample description

This cross-sectional study was conducted at the department of orthodontics at the Hospital of Stomatology, Wuhan University. The study protocol was approved by the Ethics Committees of the Hospital of Stomatology, Wuhan University (No.2022-B47). Informed written consent was obtained from patients and their parents before data collection.

We recruited consecutive children who sought orthodontic treatment and had lateral cephalograms taken for routine diagnosis from January 2022 to October 2022. The inclusion criteria for the study were (1) children aged 3 to 12 years, (2) clear identification of oropharyngeal airway and tonsils on lateral cephalograms, and (3) body mass index (BMI) below cutoff points of obesity [21]. The exclusion criteria were (1) patients with acute upper airway infection, (2) a history of previous tonsillectomy, tonsillotomy, or orthodontic treatment, and (3) craniofacial syndromes.

Within a week of the cephalometric examinations, subjects received a clinical assessment of tonsil size at the department of otolaryngology-head and neck surgery, Zhongnan Hospital of Wuhan University. The data including age, sex, weight, and height were collected for each child. Patients were divided into several subgroups with subgrouping variables of sex, age, and sagittal skeletal patterns.

### Cephalometric analysis

All lateral cephalograms were obtained with the same device (Soredex, Tuusula, Finland) and performed by the same operator, according to a standard protocol (73 kV, 10 mA). All cephalograms were taken in centric occlusion and natural head position without swallowing. The cephalograms were coded with numbers and digitized with Dolphin Imaging software (Version 11.7, Dolphin Imaging & Management Systems, Chatsworth, USA). The cephalometric measurements were analyzed by one well-trained orthodontist (C.L) with more than 8 years of clinical experience, who was unaware of each subject’s characteristics and subjective tonsil size.

**Fig. 1:**
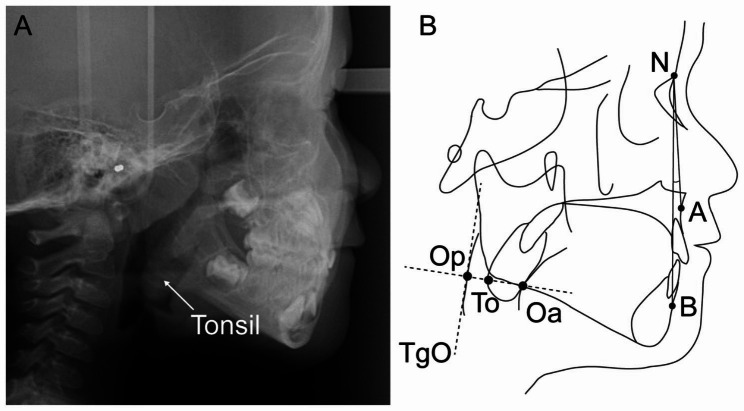
The image of the tonsil **(A)** and reference points and lines for measurements **(B)** on lateral cephalogram. N: most anterior point of nasofrontal suture; **A**: most posterior point of anterior outline of maxillary alveolar ridge; **B**: most posterior point of anterior outline of mandibular alveolar ridge; ANB: angle between N-A line and N-B line; TgO (Tangent oropharynx): tangent line to the posterior wall of the oropharynx; To (Tonsil point): the most posterior point of the posterior outline of the tonsil shadow (the nearest point to the posterior wall of the oropharynx); To-TgO (Perpendicular oropharynx): line perpendicular to TgO passing through To; Op (Oropharynx posterior): the intersection of the lines TgO and To-TgO; Oa (Oropharynx anterior): the intersection of the line To-TgO and the posterior outline of the tongue (or the anterior outline of the tonsil); T/O ratio: ratio of the distance from To to Oa and distance from Op to Oa

The reference points and lines used in the cephalometric analysis were shown in Fig. 1. On lateral cephalograms, palatine tonsils appear as an oval-shaped shadow in the oropharyngeal space close to the root of the tongue. Image enhancement brightness and contrast can be adjusted to improve image quality and anatomic landmarks for measurement. A protocol for measuring the T/O ratio proposed by Baroni et al. [5] was followed. Sagittal skeletal patterns were characterized by ANB angle: Class I (1°< ANB ≤ 5°), Class II (ANB > 5°), and Class III (ANB ≤ 1°) [22].

### Clinical assessment of tonsil size

A clinical oropharyngeal examination was carried out by an otolaryngologist (X.C) with more than 25 years of clinical experience. Patients were asked to sit up straight, open their mouths wide, and continuously pronounce the phoneme /r/. When the patient had a Friedman palate position of 3 or 4 [23] as the tonsils were not visualized, a tongue depressor was used to push the tongue against the floor of the mouth. During the examination, the investigator examined the oropharynx without activating the gag reflex, which could make the tonsils closer to the midline artificially.

**Fig. 2:**
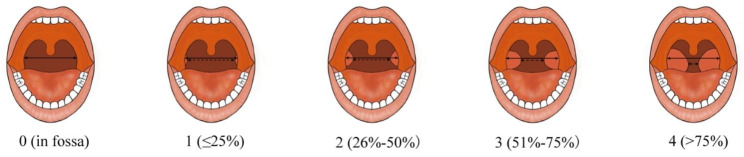
The Brodsky grading scale of tonsil size. Grade 0 (tonsils in the fossa), grade 1 (tonsils outside of the fossa and occupy ≤25% of the oropharyngeal width), grade 2 (tonsils occupy 26-50% of the oropharyngeal width), grade 3 (tonsils occupy 51-75% of the oropharyngeal width), and grade 4 (tonsils occupy > 75% of the oropharyngeal width)

The tonsil sizes of children were clinically graded on a scale of 1 to 4 according to the Brodsky scale (Fig. 2). Grades 3 and 4 were deemed a diagnosis of TH in clinical settings. Grade 0 was reserved for postsurgical patients with no tonsils who were excluded from the study. We recorded the percentage of oropharyngeal obstruction by both tonsils to assign a grade instead of assessing the left and right tonsils separately, and the same goes for the asymmetric tonsils.

### Statistical analysis

The collected data were analyzed using the SPSS software (version 26.0, Chicago, USA). The cephalograms of 30 randomly selected children were measured twice, with a 2-week interval between the measurements. Method reliability was evaluated using intraclass correlation coefficient (ICC) and measurement error was evaluated using the method of moments estimator (MME) formula [24]. Spearman’s rank correlation coefficients (ρ) were calculated to evaluate the correlation between the T/O ratio and clinical tonsil size in the total sample and all subgroups. We classified the Spearman correlations in groups of negligible (0–0.09), weak (0.1–0.39), moderate (0.4–0.69), strong (0.7–0.89), and very strong (0.9–1.0) correlation [25]. The Fisher’s z-statistics with Zou’s confidence intervals (CI) [26] was used to test for differences in coefficients between two subgroups, implemented in the software package Cocor (version 1.1.3, Duesseldorf, Germany) [27].

The receiver operating characteristic (ROC) curve was performed to analyze the validity of T/O ratios for evaluating TH. The sensitivity, specificity, positive predictive value (PPV), negative predictive value (NPV), and accuracy of different cutoff points were calculated. In all tests, P values of < 0.05 were considered statistically significant.

## Results

### Baseline clinical and cephalometric information

A total of 200 patients were assessed, and 15 were excluded from the sample due to radiographic reasons. The image of tonsils on lateral cephalograms was invisible in 11 children and obscured by the angle of mandible in the other 4 children. The final sample was thus composed of 185 subjects (101 males, 84 females; mean age 7.3 ± 1.4 years, range 3–12 years).

**Fig. 3:**
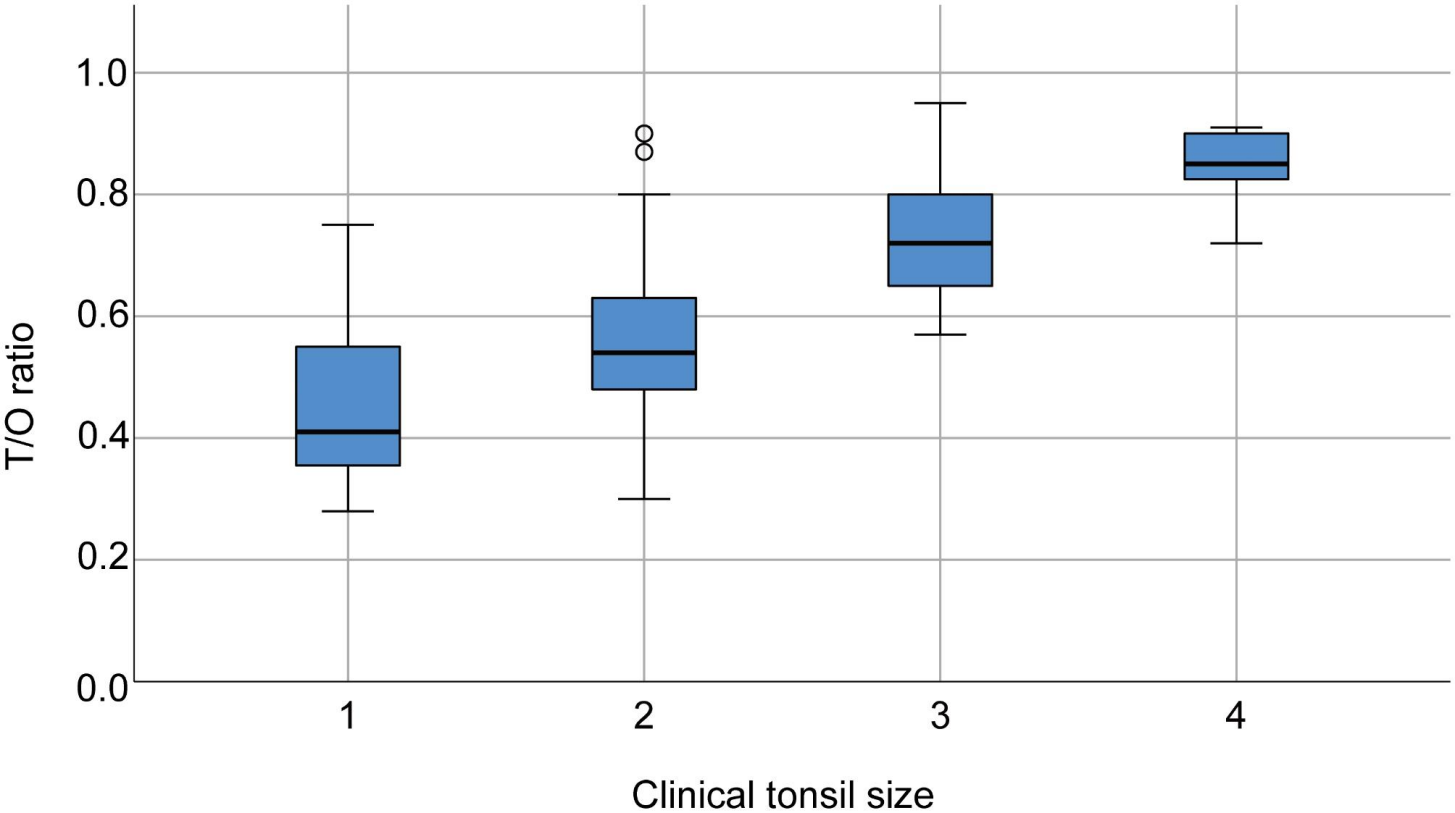
Relationships of clinical tonsil sizes and T/O ratios. Each box plot represents the median and 25th and 75th percentile. Outliers are defined by small circles

The MME error was 0.20° for cephalometric angular measurement and varied from 0.21 to 0.46 mm for linear measurements. The ICC value was 0.95 for angular measurement and varied from 0.87 to 0.96 for cephalometric linear measurements, indicating satisfactory intra-rater reliability. Table 1 showed that the majority of children had grade 2 (44.3%) and grade 3 (33.0%) tonsils, and the prevalence rate of TH (grades 3 and 4 tonsils) was 41.1% in the studied population. A cephalometric T/O ratio greater than 0.6 was present in 71 of 76 (93.4%) children with TH. The median T/O ratio of grades 1, 2, 3, and 4 was 0.41, 0.54, 0.72, and 0.85, respectively (Fig. 3). There was an overlap between the middle 50% range of T/O ratios (shown as the blue box in Fig. 3) in grade 1 and 2 groups. For some tonsils, we observed discrepancies between the T/O ratio and clinical tonsil size. One child in the grade 1 group and 7 children in the grade 2 group had T/O ratios greater than 0.72, which was the median value of the grade 3 group. Baseline information of the children with different sagittal skeletal patterns was shown in Supplementary Table 1.

**Table 1:** Characteristics and clinical data of the children participating in this study

Characteristic	Value
Age, mean (SD), y	7.4 (2.5)
BMI, mean (SD), kg/m^2^	16.9 (2.1)
Sex, N (%)	
Male	101 (54.6)
Female	84 (45.4)
Clinical tonsil size, N (%)	
Grade 1	27(14.6)
Grade 2	82 (44.3)
Grade 3	61 (33.0)
Grade 4	15 (8.1)
Tonsillar hypertrophy, N (%)	76 (41.1)

SD, standard deviation; BMI, Body mass index

### Correlation analysis between the T/O ratio and clinical tonsil size

Spearman correlation analysis showed a significantly strong correlation (ρ = 0.73; P < 0.001) between the T/O ratio and tonsil size in the total sample (Table 2). Stratifying by patient characteristics, the correlation coefficients varied from 0.67 to 0.94 in the subgroups (P < 0.001 for all).

**Table 2:** Spearman correlation coefficients between T/O ratio and clinical tonsil size in total sample and subgroups

Characteristic	N	Correlation coefficients	(95% CI)	P value
Overall	185	0.73	(0.65, 0.79)	< 0.001
Sex				
Male	101	0.71	(0.59, 0.80)	< 0.001
Female	84	0.75	(0.63, 0.84)	< 0.001
Age, y				
3–5	65	0.69	(0.53, 0.81)	< 0.001
6–8	71	0.79	(0.68, 0.86)	< 0.001
9–12	49	0.69	(0.51, 0.81)	< 0.001
Sagittal skeletal pattern				
Class I	74	0.67	(0.51, 0.80)	< 0.001
Class II	98	0.74	(0.63, 0.82)	< 0.001
Class III	13	0.94	(0.77, 0.98)	< 0.001

CI, confidence interval

Table 3 revealed that sagittal skeletal pattern was related to statistically significant differences in correlation coefficients, but age and sex were not. A significantly higher correlation coefficient (ρ = 0.94) was found in the Class III children compared with Class I or Class II children (Z = − 2.75, Z = − 2.37, respectively; P < 0.01). There was no statistically significant difference in correlation coefficients between Class I and Class II group (Z = − 0.89, P = 0.187).

**Table 3:** Correlation differences between age, sex, and skeletal pattern subgroups

	Test Statistic (z)	P value
Male group vs Female group	−0.571	0.284
3–5 group vs 6–8 group	−1.273	0.102
3–5 group vs 9–12 group	0	0.5
6–8 group vs 9–12 group	1.171	0.121
Class I group vs Class II group	−0.891	0.187
Class I group vs Class III group	−2.745	0.003*
Class II group vs Class III group	−2.369	0.009*

* a significant difference between two correlations (P < 0.05)

### The validity of cephalometric T/O ratios in the diagnosis of TH

**Fig. 4:**
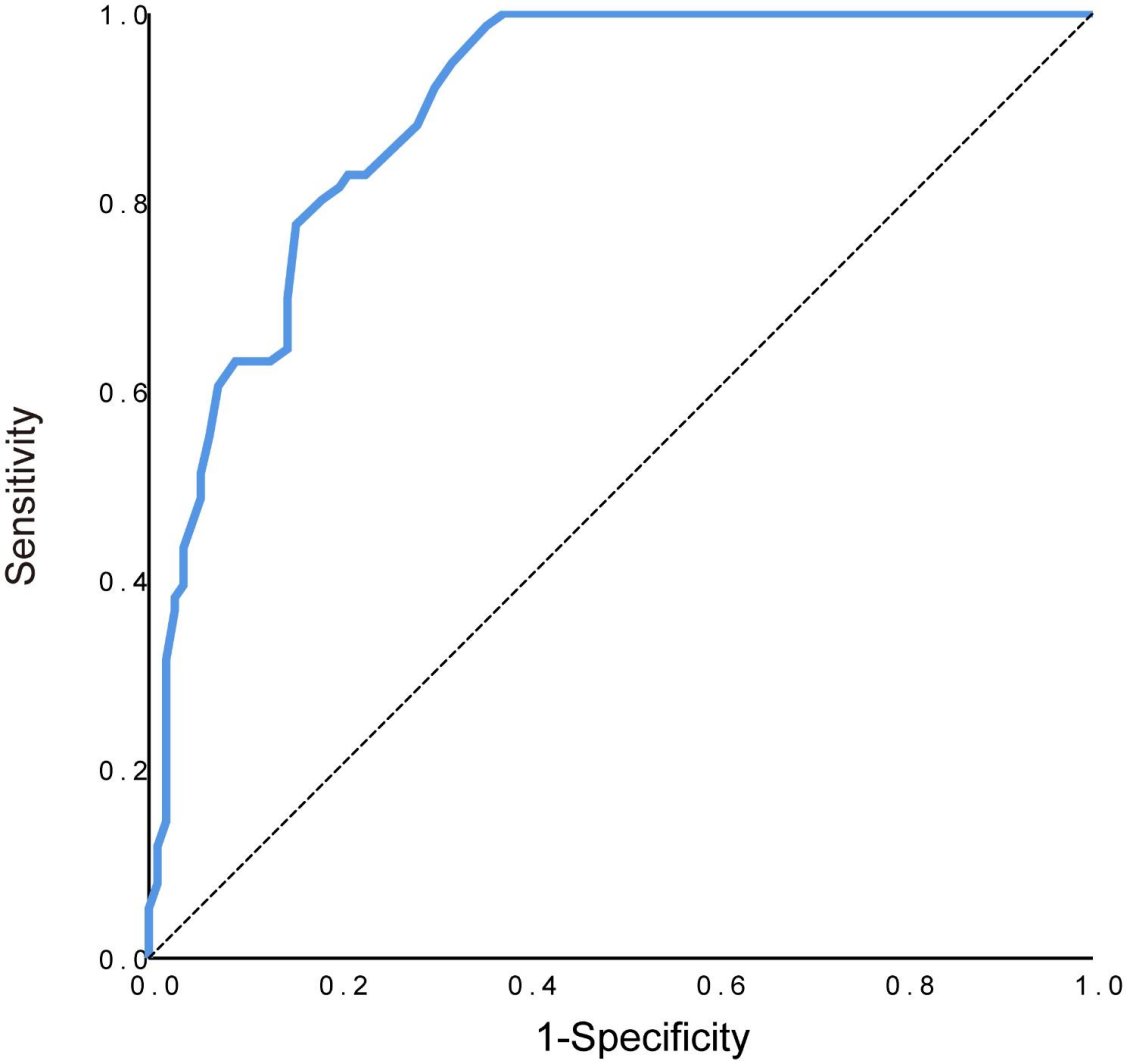
ROC curve of T/O ratios for diagnosing TH (AUC = 0.898).

As seen in Fig. 4, the ROC curve analysis revealed an area under the curve (AUC) of 0.90 (95% CI, 0.86–0.94; P < 0.001), indicating cephalometric T/O ratios demonstrated good overall accuracy in detecting TH. The optimal cutoff value of the T/O ratio for predicting TH was 0.58 (with the highest Youden’s index), corresponding to a sensitivity of 98.7% and specificity of 64.2%. Using a cutoff value of 0.5 proposed by Baroni et al. [5], sensitivity increased to 100% and specificity decreased to 45.9%. The corresponding PPV, NPV, and accuracy of the two cutoff points were shown in Table 4.

**Table 4:** Comparison of diagnostic accuracy of the different cutoff values

T/O ratio	Clinical exam		Total	Sensitivity	Specificity	PPV	NPV	Accuracy
	Positive	Negative						
Cutoff value = 0.58								
Positive	75	39	114	98.7%	64.2%	65.8%	98.6%	78.4%
Negative	1	70	71					
Total	76	109	185					
Cutoff value = 0.5								
Positive	76	59	135	100%	45.9%	56.3%	100%	68.1%
Negative	0	50	50					
Total	76	109	185					

T/O, tonsil–oropharynx; PPV, positive predictive value; NPV, negative predictive value

## Discussion

Oropharyngeal obstruction caused by TH can lead to pediatric OSA and malocclusion, so the accurate evaluation of tonsil size is a key component of clinical decision-making for both tonsillectomy and orthodontic treatment in children [28, 29]. Patel et al. [12] proposed an endoscopic grading system for quantifying tonsil size, based on the degree of oropharyngeal obstruction in both the medial-lateral and anterior-posterior dimensions. Clinicians should not focus solely on lateral obstruction and ignore sagittal obstruction because the tonsil is a 3-dimensional (3D) object in the oropharynx. Although magnetic resonance imaging (MRI) and computerized tomography (CT) could offer a 3D solution to assess tonsils [8, 20, 30], the limitations such as high costs and exposure to extra ionizing radiation confine them to routine assessments for children.

As a useful and readily accessible tool for orthodontists, lateral cephalograms can be a helpful adjunct in assessing oropharyngeal obstruction in the anterior-posterior dimension as well as predicting the clinical tonsil size. The results of the present study showed a strong correlation between the T/O ratio and clinical tonsil size in children. This can be explained because tonsils are inclined to enlarge in three dimensions rather than one dimension. This interpretation is in agreement with Wang et al [31], who found that the objective tonsil measurements including length, width, and depth were significantly correlated with clinical tonsil size. Nevertheless, the T/O ratio on lateral cephalogram sometimes may fail to quantify tonsil size because of discordances between them, especially in children with grade 1 or 2 tonsils.

Notably, the T/O ratio was more strongly correlated with tonsil size in Class III children than in Class I and II children. This may be because Class III children with larger oropharyngeal airway depth and volume [22, 32] present a more original shape of tonsils and consequently a stronger correlation, relative to Class I and II children with possible distorted tonsils. These results suggest that the T/O ratio may be more accurate in predicting tonsil size and detecting TH in Class III children. The age range (3–12 years) was chosen because it included the age at which lymphoid tissues such as tonsils reached their peak in children [1]. There was no significant difference in correlation coefficients between the various age groups, indicating the stage of development might not affect the correlation between the T/O ratio and tonsil size.

Clinically, it is more important to distinguish hypertrophic tonsils (grades 3 and 4) from non-hypertrophic tonsils (grades 1 and 2) than to predict the exact tonsil size between grades 1 and 2 or between grades 3 and 4. Our findings showed that the cephalometric T/O ratio exhibited good overall accuracy (AUC: 0.90) for evaluating TH. For a disease of TH, the ideal goal for orthodontists is to identify all patients with TH at risk of referring the occasional healthy child to an otolaryngological assessment. Therefore, it is important to maximize sensitivity (low rate of false-negative results). Although the cutoff value of the T/O ratio at 0.5 showed an excellent sensitivity of 100%, it had a poor specificity of 45.9%. The optimal cutoff value of 0.58, by contrast, had an almost excellent sensitivity of 98.7% and an acceptable specificity of 64.2%. The value of 0.58 was more reasonable to be the reference value to detect TH, as it balanced the sensitivity and specificity while maintaining almost excellent sensitivity and higher accuracy.

Employing the cutoff value of approximately 0.58, the lateral cephalogram detected nearly all subjects with TH, but it still incorrectly classified 35.8% of children. Besides, a PPV of 65.8% was acceptable but not good enough. Therefore, the potential false-positive diagnoses should be taken into consideration (lateral cephalograms may overestimate TH) when applying our findings in clinical practice. A considerable number of children in our sample were found to have hypertrophic tonsils, indicating that screening for TH among children seeking orthodontic treatment was imperative. These results could aid orthodontists or clinicians in their roles as early detectors of pediatric TH.

Most orthodontists usually pay more attention to the facial profile, teeth position, and occlusion of children and omit the clinical examination of tonsils during the first visit, as it is not a routine assessment in dental practice. Our research has validated an easy-to-use method that can provide cephalometric evidence for orthodontists to screen pediatric TH in the absence of examining clinical tonsil sizes. This can also be helpful in conducting retrospective studies that use lateral cephalograms to recognize hypertrophic tonsils, as clinical information on tonsils is often unavailable. Besides, the lateral cephalogram may offer a useful reference for otolaryngologists to predict TH when young children are uncooperative with clinical examination. However, 15 excluded children suggested that the lateral cephalogram had limitations in assessing tonsils due to its two-dimensional and static nature. The image of the tonsils can be invisible and obscured by the angle of mandible, which may lead to an unavailable or inaccurate measurement of the T/O ratio. In such cases, the clinical examination is indispensable. Therefore, it is advised to use lateral cephalograms as a screening tool for TH or an additional method to the clinical examination instead of replacing the current diagnostic criteria.

Although the clinical examination is easily performed and crucial for the diagnosis of TH, its predictive ability can be limited due to the unclear association between subjective tonsil size and OSA severity [14, 33]. Therefore, 3D evaluation and multiple measurements of tonsil size rather than a single clinical examination were recommended for children with suspected OSA. The combined use of lateral cephalogram and clinical examination may provide clinicians with a more comprehensive evaluation of tonsil size and serve as an alternative tool to 3D imaging to some extent, as lateral cephalogram adds depth to clinical examination.

### Limitation

This pilot study has a few limitations. The sample size calculation was not performed, as no similar study was in the literature. Also, the same otolaryngologist performed all the clinical assessments, and inter-rater reliability was not assessed. Additionally, due to the low proportion of Class III children in our sample and pediatric population, further studies comprising more Class III subjects are needed to form more precise results. Furthermore, future studies need to be performed to confirm the usefulness of this ratio and reinforce the validity of this preliminary cutoff value.

## Conclusions

1. Based on the present data, the T/O ratio on lateral cephalogram has a strong correlation with clinical tonsil size and exhibits good diagnostic accuracy for evaluating TH in children seeking orthodontic care.
2. Measurement of the T/O ratio may be used for the initial screening of pediatric TH when lateral cephalograms are readily available.
3. The combined use of cephalometric analysis and clinical assessment may provide clinicians with a more comprehensive evaluation of tonsil size.

### Electronic supplementary material

Below is the link to the electronic supplementary material.

Supplementary Material 1

## Data Availability

The data underlying this article will be shared on reasonable request to the corresponding author.

## Abbreviations

TH: Tonsillar hypertrophy
OSA: Obstructive sleep apnea
T/O: Tonsil–oropharynx
BMI: Body mass index
CI: Confidence interval
ROC: Receiver operating characteristic
PPV: Positive predictive value
NPV: Negative predictive value
ICC: Intraclass correlation coefficient
AUC: Area under the curve
3D: 3-dimensional
MRI: Magnetic resonance imaging
CT: Computerized tomography
SD: Standard Deviation
