# Supplementary material for: Role of the tonsil–oropharynx ratio on lateral cephalograms in assessing tonsillar hypertrophy in children seeking orthodontic treatment
Source: BMC Oral Health. 2023 Nov 7;23:836. doi: 10.1186/s12903-023-03573-z (PMC10629199; doi:10.1186/s12903-023-03573-z)
Supplement: Supplementary file 1 — Supplementary Material 1 [file 12903_2023_3573_MOESM1_ESM.docx]

Supplementary table 1. Characteristics and clinical data of the children with different sagittal skeletal patterns

|  | Class I | Class II | Class III |
| --- | --- | --- | --- |
| N | 74 | 98 | 13 |
| Age, mean (SD), y | 7.7 (2.4) | 7.1 (2.5) | 8.1 (2.9) |
| BMI, mean (SD), kg/m^2^ | 17.1 (2.2) | 16.7 (1.9) | 16.8 (2.4) |
| Sex, N |  |  |  |
| Male | 44 | 50 | 7 |
| Female | 30 | 48 | 6 |
| Clinical tonsil size, N |  |  |  |
| Grade 1 | 11 | 14 | 2 |
| Grade 2 | 35 | 42 | 5 |
| Grade 3 | 24 | 33 | 4 |
| Grade 4 | 4 | 9 | 2 |

SD, standard deviation; BMI, Body mass index.
